# The evolution of Israeli public policy for drug-using backpackers

**DOI:** 10.1186/s13584-018-0223-2

**Published:** 2018-05-04

**Authors:** Hagit Bonny-Noach

**Affiliations:** Department of Criminology, Ariel University and Board member of the Israeli society of Addiction Medicine (ILSAM), Ariel, Israel

**Keywords:** Drugs, Backpackers, Drug policy, Media, Multiple streams approach, Harm reduction, Drug treatment, Israeli society

## Abstract

**Background:**

Over the past 20 years, the young-adult backpacking trip has emerged as a significant social phenomenon in Israeli society. This has received attention from scholars specializing in anthropology and tourism research, but only a few analytical studies exist on the drug policy processes and few provide Israeli social and health perspectives. The interaction of policymakers, media, and health deviancy is an important focus of inquiry. This study charts the establishment of a drug policy for Israeli backpackers. It covers the period from the emergence of the problem in the early 1990s until the present.

**Methods:**

This study employs content analysis of newspaper articles and official documents, protocols, and reports written by policymakers and professionals. The latter were mostly produced by the Israel Anti-Drug Authority (IADA) and the Special Committee on Drug and Alcohol Abuse (SCDAA) in the Israeli Knesset. These are the two major Israeli agencies responsible for drug policy.

**Results:**

Three periods in the establishment of backpacker drug policy can be identified. *First period* – until late 1995: No drug problem was recognized. The subject was not part of the public agenda. Even so, many backpackers were actually taking drugs. *Second Period* – late 1995 to 2000: The Israeli media started to report intensively on backpacker drug use. The issue then flared up into a significant ‘social problem’ demanding health and social solutions. In this phase, policymakers capitalized on a window of opportunity, and formulated a policy emphasizing prevention. *Third period* – from 2001 until the present: A sea change in institutional attitude occurred. In this period, drug-policy emphasis shifted from prevention to therapeutic-treatment approaches. As a result, harm reduction and unique treatment strategies were developed.

**Recommendations:**

Policymakers should continue to improve health prevention, treatment, and harm reduction resources. It is recommended that the Ministry of Health set up consultation centers at clinics for travelers. These would provide support and assistance to backpackers before, during, and after their trips.

The attention that Israel’s drug policy for backpackers gives to prevention, treatment, and harm reduction is the first of its kind and unique. It can therefore serve as a model for other countries.

## Background

The backpacking trip is practically institutionalized in Israeli society as a young-adult rite of passage [1–4]. This social phenomenon emerged over the last several decades, and trips to Asia or South America are considered part of the typical life route of Israelis after mandatory army service [5]. Approximately 50,000 young Israelis go on backpacking trips each year [2, 6, 7]. As a rite of passage [8, 9], these trips are a way to disconnect from societal demands, and they represent a challenge to established values and norms [1, 5, 9–11]. However, foreign backpackers are exposed to various health-related physical and mental risks, including injuries, diseases (e.g., sexually transmitted infections), and more [7, 12, 13].

Drug consumption is a relatively common reported backpacking activity among Israelis, Australians, and several Western European countries (including the United Kingdom, Germany, Italy, and Sweden) [7, 11, 14–17]. For Israelis, high rates of drug usage are reported in comparison to the general population and their non-backpacking peers [1, 15, 18, 19]. In fact, a primary Israeli motivation to visit India is to experience drugs [9]. The most commonly used drugs are cannabis-based products (e.g., ganja- Indian marijuana and hashish- Indian jaras), hallucinogenic mushrooms, cacti, ecstasy, and LSD [1, 2, 6, 11, 17]. A real danger exists for Israeli backpackers who use drugs; they may suffer mental and physical damage due to abusive habits. Hundreds of backpackers have been seriously affected by mental illness, including first-episode psychosis, acute psychosis in different degrees of severity, and dual diagnosis due to drug use. Hospitalization in psychiatric wards may be required [1, 2, 6, 7].

The Israeli backpacking culture has attracted anthropological, sociological, and tourism studies research [3, 5, 9, 11, 20], with a focus on drug use behaviors [1–4, 10, 11, 20].Unfortunately, other important perspectives are lacking and some important aspects of the phenomenon have been analyzed. For instance, the fusion of Israeli policymaker and media responses with social perception of behavior as a health risk and deviancy remains to be studied [1, 21].

This study tracks the establishment of a backpacker drug policy from the formative early 1990s until the beginning of the twenty-first century. It focuses on how Israeli backpackers using drugs developed into a social problem and how a corresponding drug policy was formed.

Definitions of *drug policy* range from ‘all activities related to illicit drugs’ to ‘a set of principles or an ideology that directs public action in this field (e.g., war on drugs, harm reduction, and more)’ [22]. The theoretical framework of this study is based on political scientist, John Kingdon’s Multiple Streams Framework [23–25]. Kingdon devised a threefold approach to account for policy formation in the American political landscape. His framework has been instructively applied to other political contexts such as European Union policy analysis.

For Kingdon, the agenda setting process is influenced by three “streams” which at times interact to produce “windows of opportunity”. Usually, the problem, policy, and political streams flow along different channels. They remain independent until a specific point in time when a policy window opens, and then the streams cross. *The problem stream* refers to issues that capture attention. These problems are seen as public in the sense that government action is needed to resolve them. *The policy stream* represents the output of experts and analysts who examine problems and propose solutions. This may be conceptualized as a “policy primeval soup”, in which policy ideas and solutions are formed, developed, rejected, and selected. *The political stream* refers to factors that influence the body politic. These include public opinion, swings in national mood, election results, and interest group advocacy campaigns.

As such, this study is based on the following questions: How did Israeli backpacker drug use develop into a social problem needing social and health policy solutions? What is the media’s role in the construction of a corresponding drug policy? Which interventions did policymakers include in their drug policy? What ideologies direct public and policymaker action in this field? Finally, how does the Multiple Streams Approach clarify this drug policy formation and change?

## Methods

This study uses content analysis. This is a flexible method for analyzing text data to provide knowledge and understanding of phenomena. It also elicits subjective interpretation of content through systematic classification, coding, and identification of themes or patterns [26, 27].

Data was collected from hundreds of newspaper articles, mostly from the three popular national newspapers (Yedioth Aharonoth, Maariv, and Haaretz). Most of the sampling included print articles and was gathered from IFAT, a leading media information company that works for IADA and collected drug-related information in Israel from all media agencies. Newspaper articles were also gathered from the Knesset archives, universities, and periodicals library in Bet Ariella, an archive of Israeli press coverage. In total, our study utilized 93 newspaper articles.

Official documents, protocols, and reports written by policymakers and professionals were analyzed, mainly from 1996 to 2006. These stem mainly from the two major agencies responsible in Israel for drug policy: the Israel Anti-Drug Authority (IADA) and the Special Committee on Drug and Alcohol Abuse (SCDAA) in the Israeli Knesset [28, 29]. The first is the central institution responsible for mobilizing all government organizations and public authorities in the enforcement, treatment, and prevention of drug use. The second is the committee supervising all authorities that deal with drug abuse in Israel. Hundreds of protocols and reports can be found in the archives of IADA (e.g., the Backpacker Project) and the Knesset. Some SCDAA protocols were found on the Knesset website (online protocols are available from 2001). This study is based mostly on an analysis of 24 SCDAA protocol documents.

## Results

Three periods can be identified in the establishment of a backpacker drug policy:

### First period – Until late 1995: Backpacker drug use does not constitute a ‘social or health problem’

During this period, no backpacker drug problem was recognized. Led by IADA, it was assumed by Israeli policymakers that only marginal and non-threatening levels of drug usage existed. They perceived drug use as uncharacteristic of Israeli backpackers [1, 30]. Global backpacker drug use had been acknowledged for many years [14, 31]. During the 1980s, young Israelis began to backpack [5], although almost no studies focused on drug use during this period. So, it remains unknown when exactly drug use began to be widespread. The same is true of its extent, and trends exhibited during these years [1].

The social activity of raves (i.e., all night techno-music outdoor beach parties) contributed to drug use escalation [1, 30, 32–34]. These were especially common for backpackers in the Indian state of Goa [35] and on islands in southern Thailand [16]. However, it is assumed that Israeli backpackers engaged in recreational drug use from the very beginning of backpacking culture in the early 1970s. These habits escalated and were apparently already widespread during the mid-1980s [1, 8].

Despite drug proliferation during this period, general unawareness was still the norm. The Israeli policymaking establishment, the media, and parents of the backpackers were ignorant of the extent of the problem. They did not really know what happened on a typical backpacker trip [1]. Of course, no internet or cell phone communication then existed. Thus, the only information came from written letters and the occasional call home. To some degree, lack of awareness derived from backpackers themselves keeping drug-use a relatively well-kept secret from adult authority figures. However, it was well known to backpacking peer groups and Israeli youth. Descriptions of backpacker antics were soon heard in Israel, along with photographic evidence of overseas drug experiences [30, 33]. Some treatment professionals, in private clinics or psychiatric hospitals, treated backpackers who suffered from mental disorders related to drug abuse. Yet the prevailing notion was that drug use was uncommon. It was not a social problem and did not need a formal drug policy [1, 36]. Some articles on backpacker drug behaviors were occasionally published in Israeli media outlets. These highlighted backpackers who fell victim to drug abuse overseas. For instance, “a 30 year-old man that ate hallucinogenic mushrooms in Thailand was returned to Israel for hospitalization” [37]. Others offered lurid glimpses into the backpacking experience. One article was titled, “Everything you wished you knew about acid and didn’t dare ask” [38]. Here, the journalist focused on backpacker drug use in Thailand. Yet these random articles failed to arouse significant social interest beyond simple curiosity [1].

The subject of backpacker drug abuse was raised for the first time in Israel on June 21, 1993 at the end of the SCDAA in the Knesset. The initial pretense was a discussion of raves and acid parties in Israel [39]. Young adult backpackers were first mentioned as a group that had imported this trend into Israel. The use of drugs among backpackers was mentioned, but did not draw attention.

The subject was raised again on February 28, 1995, without attracting concern. This SCDAA gathered to discuss the subject of student drug use based on an article in *Pi Haton* (a student newspaper of the Hebrew University of Jerusalem) [40]. The article described widespread student drug use during a pre-university backpacking trip. Explicit statements from editors of the student newspaper and other young student representatives were made to the committee. However, the data was rejected.

A third SCDAA meeting on July 11, 1995 focused on young Israelis engaged in cannabis smuggling in the Sinai. This discussion also made reference to backpacker drug use in India [41], but only towards the end of the meeting. Therefore, it was agreed that an exclusive discussion of drug-using backpackers would take place the following week. As a result, the SCDAA officially convened to discuss the subject of “The growing phenomenon of Israeli young people who consume hard drugs in India and the Far East” [42]. The committee included professionals and came to realize that the percentage of drug-using young adult backpackers remained unclear. It was decided not to rush into implementing a policy. A more thorough investigation and clarification of the facts needed to be carried out before publicizing the issue.

### Second period – Late 1995 to 2000: The media started to report intensively on backpacker drug use. As a result, a drug policy dedicated to finding health and social solutions was demanded

At the end of December 1995, an onrush of backpackers flooded the beaches of Goa for New Year celebrations. The Israeli media then started to report intensively on backpacker drug use. They exposed the public to the supposed ‘facts’ of drug abuse, mental illness, and even death. It was reported that “thousands of ‘salt of the earth’ Israelis [were] getting high on the beaches of Goa.” Sixteen articles were published by newspapers between 26 December, 1995 and 1 January, 1996. This included long form weekend articles. News reporters were sent to Goa to cover backpacker drug use. They used their journalistic resources such as graphic photographs, lengthy articles, and imposing headlines. They also employed morally and emotionally loaded language.

Table 1 displays the examples of Israeli newspaper headlines on various issues such as correlation of backpackers and drug abuse, delinquency and drug trafficking, and mental illness, insanity, and death. It also displays media calls for parents and policymakers to take action.

**Table 1:** Examples of headlines in Israeli newspapers

Issue	Israeli newspaper headlines:
Correlation of backpackers and drug abuse	“Thousands of young Israeli participants at drug and alcohol parties in India,” Ma’ariv, December 26, 1995. By Yoav Limor; “Thousands of Israelis for seven days dropping acid at huge beach party in India,” Yediot Aharonot, December 29, 1995. By Asefa Peled; “Indian trip: Yes, thousands of Israelis participate each year in acid parties in India,” Ma’ariv, December 29, 1995. By Yoav Limor; “Israelis get high in India: Taking drug-trips,” Yediot Aharonot, January 1, 1996. By De Bar, S; “Thousands of Israelis at the biggest drug party in the world,” Yediot Aharonot, December 26, 1995. By Peled, A.; “Sylvester in Goa: The biggest drug party. Israelis in Goa celebrate tonight,” Ma’ariv, December 31, 1995. By Capra, M.; “Goa: Israelis, drugs, and Sylvester. A few hours before the opening of the biggest drug party in India,” Ma’ariv, January 1,1996. By Capra, M.; “Sylvester in Goa: Mom, what a great trip! A quarter to seven in the morning the sun rises in Goa and the drug party reaches its peak,” Ma’ariv, January 2, 1996. By Capra, M.; “Goa,” Ma’ariv Weekend, January 5, 1996. By Capra, M.
Correlation of backpackers, drug abuse and mental illness and insanity	“Five Israelis who took hallucinogenic drugs in India suffer from psychotic episodes: Possibility of sending a plane to India to rescue victims of drugs,” Ma’ariv, December 27, 1995. By Yaakov Galanti; “24-year-old Israeli woman rescued from Goa in difficult psychotic condition,” Yediot Aharonot, February 19, 1996. By Sofer, R.; “Israeli who smoked drugs in Goa admitted to psychiatric hospital,” Ma’ariv, February 2, 1996. By Capra, M.; “Israeli woman rescued from Goa,” Ma’ariv, February 19, 1996. By Shaked. Y.
Correlation between backpackers, drug abuse and death	“The visit to the East almost finished his life: A young man boy tells his mother he took magic mushrooms, but after returning to Israel, suffered a severe reaction and was hospitalized,” Ma’ariv, December 27, 1995.
	“For David Kim, Goa is a bad dream: a year ago rescued…,” Ma’ariv, December 27, 1995. By Golan Yosifon; “Return from Goa,” Yediot Aharonot Weekend, January 5, 1996. By Argaman-Barnea, A.; “Celebration ended in Goa,” Yediot Aharonot, January 16, 1996. By Baum, A.& Eshel, S.; “The solution is not India! Instead of necessary treatment, unit sent to Goa beach, with or without condoms, wrangles with authorities,” Haaretz, January 1, 1996. By Golan, A
Correlation between backpackers and delinquency/ drug trafficking	“Israeli backpacker arrested in Goa suspected as drug dealer,” Ma’ariv, January 10, 1996. By Brenner, D. & Cohen, A.; “30 young Israelis arrested in India and Thailand for drug offenses,” Haaretz, January 24, 1996. By Alon, G. & Zrahiya, T.
Correlation of construction of clear and tangible danger due to these behaviors and media prediction of dire consequences if failure to act: Creates call for parents to worry over their children’s potential interaction with drugs	“Possibility of sending plane to India to rescue victims of drugs,” Ma’ariv, December 27, 1995. By Yaakov Galanti; “Israelis in India panic: Hundreds of worried parents phone Goa, begging children to go home,” Yediot Aharonot, December 31, 1995. By Asefa Peled; “Parents asked to fly to Goa, India, to return their children from drug party,” Haaretz, December 27, 1995. By Rali, S; “Parents do something about children traveling to India! Young Israeli Limor Cohen pleads in emotional letter to newspaper,” Yediot Aharonot, December 31, 1995. By Rimon, N.
Correlation of reference to State of Israel (including law enforcement) and its actions in addressing the serious problem of drug using backpackers	“Police considering sending narcotics detectives to Goa beach,” Yediot Aharonot, December 28, 1995. By Meiri, D., Travelsi-Hadad, T. & Baum, I.; “Indian embassy prepares list of Israelis in Goa zone,” Yediot Aharonot, December 29, 1995. By Meiri, D. & Yakir, Y.; “Appointed committee for Israeli drug problems abroad,” ITIM (Israel News Agency), January 8, 1996; “Tightening cooperation between Israeli and Indian police forces: Israeli police representatives may go to India to investigate drug parties,” Haaretz, December 28, 1995. By Shapira, R.; “Police afraid of pressure in ‘Goa affair’: Indians may arrest Israelis,” Haaretz, January 3, 1996.

According to Kingdon’s three streams [23], the first phase constitutes the problem stream. In our case, the media dominated this stream by defining drug-using backpackers as a problem that potentially required attention. As such, it could now be seen as a political matter. Attention attraction is a major achievement, which must be acted on quickly before attention shifts elsewhere. Only a few problems can reach and occupy the top level of any given policy agenda. This is partly due to the powerful competition for attention [23, 24].

As noted, information on the topic was scarce. The formal system in place for the supervision of drug use in the State of Israel was not yet prepared. This was a new problem previously judged to be marginal [36, 42]. In fact, policymakers tend to have ambiguous aims and problem-solving approaches. In contrast, interested actors are more driven to quickly research options and produce viable solutions [24].

From the Multiple Streams Approach [23, 24], this can be seen as ushering in the second policy stream. This pertains to the many potentially available policy solutions to a set of problems. The policy stream originates with communities invested in policy solutions such as intellectuals, professionals, bureaucrats, and interest groups in various fields.

While attention lurches quickly from issue to issue, viable solutions involving major policy change take time to develop. To deal with disconnect between fickle attention and slow policy development, policymakers develop widely accepted solutions in anticipation of future problems. They then find the right time to exploit or focus attention on a relevant problem [23, 24]. In the case of drug-using backpackers, policymakers failed to predict this specific problem and did not have prepared solutions ready to deploy.

In the attempt to control the situation caused by media pressure, a quick and arguably rash solution was proposed by the Director General of IADA. Concerned parents with sons and daughters in India would be sent on a chartered flight to accompany their children back to Israel. However, this proposal was published in newspapers before it was officially authorized. Ultimately, only a few parents responded to the offer. The proposed chartered flight never materialized [1, 36]. Only after this first aborted solution did policymakers start to devise new solutions to this problem.

A SCDAA meeting took place on January 23, 1996, at the height of media coverage. This time, the subject was “The growing phenomenon of Israeli young people who consume hard drugs in India and the Far East (Drug parties in Goa and going to temples in India)” [43]. In this committee, policymakers claimed that drug use in the younger generation was symptomatic of escapism, lack of social direction, and absence of values. Also, the committee denounced media exaggeration of the phenomenon. It was decided that IADA would find relevant solutions to these social and health problems.

At that point, policymakers were aware of the existence of a moral-social-health problem. Yet it was not at all clear if the situation described in the media was authentic, fabricated, or exaggerated [1]. Thus, in the early stages of IADA’s formulation of a drug policy and solutions (in 1996), it was still important to confirm its objective proportions. Data needed to be validated and more questions asked: Which drugs were being using? Where were they using them? [1, 36].

Correct data is required for the creation of a good drug policy. IADA professionals began to encourage research and survey studies to elicit more data on rates of backpacker drug use [36]. In these research endeavors [10, 19, 33, 36, 44, 45], it became apparent that drug use rates were indeed as high as the media reported. According to IADA, a drug policy was justified, with activities and allocation of funds targeting the backpacker population.

The issue was now taken seriously and remained on the public agenda in several arenas. The first and dominant arena was the ***institutional arena***, led by IADA. They established the ‘Backpackers Project’ in mid-1996, emphasizing prevention and awareness. This included providing medical and legal information on the dangers of drug use, prevention tools, and research activities. Pamphlets were distributed containing relevant medical and legal information to traveler clinics at the Ministry of Health [1, 36]. Even though Goa was the locus of media incitement, the ‘Backpackers Project’ encompassed most Asian and South American backpacker destinations. The Foreign Ministry also established a special department to address drug problems in the backpacking community working in collaboration with IADA [11, 36].

In the ***media arena***, the issue continued to influence the public agenda. During this period, media coverage of the issue became routine. Sample headlines included, “A message from the Beer Yaakov (mental hospital) isolation ward: ‘Don’t mess with drugs and hallucinogenic mushrooms in Goa’” [46]; “Conspiring against Israeli backpackers in Goa” [47]; and “Israeli receives 10-year prison sentence in India” [48].

During this period, the issue also gained momentum in the ***academic arena***. Researchers showed significant interest in IADA’s initiatives, career academics and graduate students alike [44, 45, 49]. However, there was still no official response for the treatment of backpacking drug-abuse victims. They were treated by private psychiatrists and psychologists or in mental hospitals.

### 1.3 Third period – 2001 until the present: Drug policy changed from prevention to harm reduction. The focus was now on specific therapeutic-treatment approaches for backpacking victims of drug abuse

During the third period, the attitude of the Israeli establishment and general public began to soften a bit. It was recognized that young adult backpacker behavior could not be completely controlled [1, 36]. Thus, the problem came to be defined in therapeutic-treatment terms. The solution now focused on treatment of drug-abusing backpackers perceived as the central core of the problem [36]. Drug policy shifted to unofficial focus on harm reduction, providing tips for backpackers, and information on what to do in case of emergencies such as acute psychosis due to substance abuse. This change in perspective was expressed through the development of new responses, as evidenced by two unique frameworks. First, in 2001, *Kfar Izun* (“Harmony Village”) was founded in Israel for rehabilitating backpackers from acute psychosis due to substance abuse. Second, in 2003 the ‘Israeli Warm Home’ was established in India, an open house providing an information resource center and first response for those negatively affected by drug use [2, 50]. The Israeli Warm Home was a partial attempt to treat young backpackers with a harm reduction approach. Although IADA supported a complete ban of illicit drugs [28], the Israeli Warm Home implicitly recognized the improbability of entirely preventing backpacker drug use [2, 50]. Additionally, in recent years health insurance agencies in Israel have crafted policies to include rescue and airfare provisions for backpackers affected by drug use [2].

During this period, the issue remained on the public agenda in several arenas. In the once dominant ***institutional arena*** led by IADA, an attitude shift took place, and the Backpackers Project was shuttered in 2006. However, both *Kfar Izun* and the Israeli Warm Home continued to receive support. In 2008, IADA published *Backpackers and Drug Abuse: A Documentary, Research, Treatment and Prevention Perspective*. This book is the first document of its kind. It consists of a mix of 26 chapters on backpacker descriptions of their subjective experiences and scientific research in the field from academia, therapists, and professionals [1, 36, 51]. In addition, workshops were held for young soldiers prior to their release from the military. These focused on inherent dangers of drug use and long-term consequences [2]. In the ***institutional***-***political arena***, the issue remained relevant. Most political activity revolved around committee meetings of the SCDAA in which the problem continued to be debated [52–54]. A special department was established in the Foreign Ministry to continue to address this problem as well as the ***media arena,*** which continued to publish articles on a regular basis.

## Discussion

This study shows how the issue of drug using Israeli backpackers evolved into a social problem that needed social and health policy solutions from the early 1990s until the start of the twenty-first century. As noted, this phenomenon can be categorized into three periods. In the first stage, no problem was formally recognized. In the second stage, the problem was fanned largely by the media. On the one hand, the Israeli press was opportunistic in chasing after the best scoop on a new and lurid story. On the other, they operated out of moral concern for the fate of Israeli society. They exposed the “dark heart” of backpacking culture. Societal values and behavioral norms were questioned.

In a Multiple Streams Approach [23, 25], drug-using backpackers can be seen as a topical problem issue. It then emerged as prominent on the policy agenda. Thus, the three streams of problem, policy, and politics capitalize on a window of opportunity during a critical point in time. The problem stream was dominated by the media. They defined drug-using backpackers as a social problem in urgent need of political intervention. The policy stream represents potential policy solutions that originate with communities of policy solution professionals. These include intellectuals, bureaucrats, and specialists. The third stream of politics is populated by factors motivating the government (such as the resulting governmental chaos from Rabin’s assassination). These include change in national mood, elevation of the problem on the public agenda, and formulation of drug policy based on backpackers.

As noted, the last stream is characterized by policymakers motivated by opportunity to turn solutions into policy [24]. Accordingly, change of government provides both motive and opportunity. Policymakers consider factors that set the national mood and feedback they receive from the media and public. After the media coverage, the issue of backpacker drug abuse was taken seriously by Israeli society and policymakers. They demanded a drug policy dedicated to finding health and social solutions. This was at first formulated around prevention (during the second stage). As policy measures were implemented in the third stage, it evolved into emphasis on treatment and harm reduction.

Harm reduction is a general term for pragmatic interventions aimed at reducing problematic behaviors. However, complex ethical dilemmas arise from these practices [55]. In Israel, there is some controversy regarding harm reduction as applied to illicit drug use. It has some legitimacy in specific domains such as heroin addiction. For instance, this perspective informs syringe exchange programs and substance substitution such as Subutex (buprenorphine) and Methadone. However, it is generally not considered acceptable for “normative young adults” engaging in illicit drug use. Therefore, it is somewhat surprising that policy makers adopted such an approach for young backpackers.

How did it transpire that this population should be treated differently than “regular” drug users? Why did drug policy towards backpackers undergo a transformation from prevention to harm reduction? To understand the policy formation process, general political and institutional contexts need to be considered [56]. Social and cultural factors played important roles in influencing drug policy*.*

The most plausible account is class-based. *Backpackers, policymakers, and media representatives come from the middle classes and social elite* [11]. Many backpackers travel after military service, before university studies, or during semester breaks. Young backpackers are considered normative, conformist, and productive members of society. Many return to universities and colleges without presenting an urgent social problem. In contrast, drug addicts constitute a negative societal influence. The Israeli stigma of drug use as deviance (particularly regarding heroin addicts or “junkies”) does not seem to apply to backpackers.

Most heroin addicts belong to the lower socio-economic classes and often finance their addiction through crime. The social circumstances of trekking may encourage drug use. But for the most part, this usage is temporary, random, and experimental. That is, they are normative post-army youth in a liminal life stage [57] taking time off from their conventional life-path [58]. They ultimately return home unharmed, stop using drugs, and continue an acceptable routine career trajectory. They engage in academic studies, integrate into the workforce, and raise families. As noted, policymakers and media professionals belong to the middle and upper-middle classes. Significant numbers of their children were or will be backpackers. This may likely contribute to unwillingness to stigmatize the backpacker population as drug-abusing deviants. The establishment treated them with tolerance, as self-exploring young people. This hiatus stage of drug use constitutes a quasi-legitimate rite of passage and part of the maturation process.

In addition, backpacker drug-use takes place *Out-of-country.* The usual intervention model regarding drugs is one of ‘supply and demand reduction’ [59]. For policymakers, combatting drug use means addressing both demand and supply. However, the State of Israel has no control over supply in destination countries, many of which offer cheap and easy access to drugs. It is even claimed that local permissiveness of drugs is a means of economically enhancing the tourist industry. Thus, intervention must focus on demand reduction; that is, encouraging them to stop consuming (“demanding”) drugs. Indeed, drug policy is a field in which issues of public order and public health often conflict [56]. In this case, drug policy focuses on backpacker health and cannot feasibly deal with public order abroad. This may have led to the adoption of a harm reduction approach as established in India in the ‘Israeli Warm Home’. Finally, it is noteworthy that *in professional treatment, prevention begins as early as possible*. However, this population of young people is defined as beyond the stage of preventative intervention. Therefore, IADA did not invest large sums of money in the ‘Backpacker Project’ as it was working primarily on promoting universal information and prevention. Most prevention efforts continue to be invested in children and youth.

In sum, this study extends knowledge of drug policy formation and its establishment, with a focus on the backpacker community. In Israel, the major agencies tasked with dealing with the social problem of Israeli backpacking drug use implemented a drug policy with significant social and health implications.

## Conclusion and recommendation

Israeli policymakers should continue to develop and improve drug policy based on drug-using backpackers. They should include health prevention, treatment, and harm reduction aspects. Focus on treatment and harm reduction should be supplemented with greater emphasis on prevention – before and during the trip.

This study highlighted the reactions of the two major agencies responsible in Israel for drug policy: IADA and SCDAA. But there are more stakeholders that should be involved in backpacker drug policy formation. These include the Ministry of Health, Ministry of Foreign Affairs, Ministry of Education, and Health Funds. In addition, medical and non-medical professional associations as well as academia should not be overlooked. For example, the Ministry of Health should set up consultation centers in clinics for travelers to provide support and assistance to backpackers before, during, and after their trip. They should focus on general backpacker health risks, including using drugs.

Finally, many young backpackers from other countries also engage in drug use [15, 16, 19]. Other national models may benefit from developments in Israeli policy. Israel’s focus on prevention, treatment, and harm reduction is a unique model. It is the first of its kind in the world and can serve as an initial guide for other countries. Policymakers in Israel should consider collaboration with their counterparts from other countries. This can lead to greater learning about the scope of the phenomenon, identifying risk groups among backpackers, and collaboration in possible interventions.

## Abbreviations

IADA: Israel Anti-Drug Authority
SCDAA: Special Committee on Drug and Alcohol Abuse
